# Differential serotonin transporter (5‐HTT) and 5‐HT_2_ receptor density in limbic and neocortical areas of adults and children with autism spectrum disorders: implications for selective serotonin reuptake inhibitor efficacy

**DOI:** 10.1111/jnc.14832

**Published:** 2019-10-27

**Authors:** Cheryl Brandenburg, Gene J. Blatt

**Affiliations:** ^1^ Program on Neuroscience Hussman Institute for Autism Baltimore Maryland USA

**Keywords:** anterior cingulate cortex, autism, selective serotonin reuptake inhibitors (SSRIs), serotonin receptors (5‐HT2, 5‐HT1A), serotonin transporter (5‐HTT)

## Abstract

As selective serotonin reuptake inhibitors (SSRIs) are among the most commonly prescribed medications in autism, we aimed to determine whether targets for SSRIs are differentially affected in three cortical areas in children and adults with autism compared to neurotypical individuals. Utilizing a large cohort of postmortem brain tissue (*n* = 14–19 per group), saturation ligand binding assays were conducted on sections from the anterior cingulate cortex (ACC), posterior cingulate cortex, and fusiform gyrus (FG). Specific binding to the 5‐HT transporter (5‐HTT) as well as to 5‐HT2 and 1A receptors (5‐HT₂, 5‐HT_1A_) was quantified in superficial and deep layers of each region using the ligands [^3^H]‐citalopram (5‐HTT), [^3^H]‐ketanserin (5‐HT_2_), and [^3^H]‐8‐OH‐DPAT (5‐HT_1A_). A Welch’s *t*‐test was utilized to compare receptor densities (*B*_max_), revealing a statistically significant decrease in 5‐HTT within the ACC of the entire autism cohort. There was also a decrease in 5‐HT_2_ receptor density in the ACC in the adult cohort, but not in child postmortem autism cases as compared to controls. Comparing linear regression lines of *B*_max_ values plotted against age, shows a significantly lower intercept for 5‐HTT in autism (*p* = 0.025). 5‐HT₂ density increases with age in control cases, whereas in autism there is a decrease with age and significantly different slopes between regression lines (*p* = 0.032). This suggests a deficit in 5‐HTT within the ACC in individuals with autism, while decreases in 5‐HT₂ density are age‐dependent. There were no differences in receptor densities in the posterior cingulate cortex or FG in autism and no differences in ligand affinity (*K*_D_) across all regions and ligands examined.

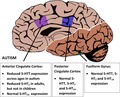

## Graphical abstract

Although selective serotonin reuptake inhibitors (SSRIs) are among the most commonly prescribed medications in autism, several studies show variable efficacy with SSRI use. Some of this variability may be a result of differential expression of serotonin receptors across individuals. The objective of this study was to determine differences in density and/or affinity of the serotonin transporter (5‐HTT), serotonin 2 receptor (5‐HT_2_) and serotonin 1A receptor (5‐HT_1A_) between autism and neurotypical individuals through saturation binding assays within three cortical areas. Our findings support the growing evidence for caution when administering SSRIs to children, while adults may benefit from these treatments.

**Abbreviations used**
^3^H: tritiated
5‐HT: 5‐hydroxytryptamine (serotonin)
5‐HT_1A_: serotonin 1A receptor
5‐HT₂: serotonin 2 receptor
5‐HT_2A_: serotonin 2A receptor
5‐HTT: serotonin transporter
ACC: anterior cingulate cortex
ADHD: attention‐deficit/hyperactivity disorder
ADI‐R: autism diagnostic interview‐revised
ASD: autism spectrum disorders
*B*_max_: receptor density
CDC: Centers for Disease Control and Prevention
FG: fusiform gyrus
fMRI: functional magnetic resonance imaging
*K*_D_: equilibrium dissociation constant
nCi: nanocuries
PCC: posterior cingulate cortex
PET: positron emission tomography
PMI: postmortem interval
SLC6A4: serotonin transporter gene
SPECT: single photon emission computed tomography
SSRIs: selective serotonin reuptake inhibitors

Peripheral hyperserotonemia was the earliest demonstrated neurochemical change in individuals with autism spectrum disorders (ASD) (Schain and Freedman 1961) and has since become the best replicated biomarker, as recent meta‐analyses conclude that hyperserotonemia is present in 25–45% of the autism population (Gabriele *et al.* 2014; Chen *et al.* 2017; Eissa *et al.* 2018). Furthermore, whole blood maternal serotonin levels during pregnancy are associated with altered cognitive abilities and core neurodevelopmental outcomes in offspring with ASD (Montgomery *et al.* 2018).

In the last decade, attention also has been directed toward the study of central serotonin levels as serotonin (5‐HT) plays important roles in neurogenesis, cell migration, synaptogenesis, plasticity, 5‐HT transporter (5‐HTT) function, and signalling during brain development and maturation, as well as a variety of other neural processes (for review see: Garbarino *et al.* 2018). The usage of drugs targeting 5‐HTT has been increased in an effort to treat symptoms of depression and/or other core symptoms in a variety of neuropsychiatric disorders. In a Centers for Disease Control and Prevention report, between 2011 and 2014, 12.7% of persons over 12 years of age had used antidepressant medications in the previous month, 68% took these medications for 2 years or more, while 25% took them for 10 years or more (Pratt *et al.* 2017).

Selective serotonin reuptake inhibitors (SSRIs) have been effective in treating patients with major depressive, anxiety and obsessive compulsive disorders and have been widely prescribed to individuals with autism for the treatment of repetitive behaviors (King and Bostic 2006), as 21–32% of chlidren with ASD are prescribed one or more antidepressant medications (Langworthy‐Lam *et al.* 2002; Aman *et al.* 2003; Oswald and Sonenklar 2007). Pharmacotherapeutic treatment for individuals with autism has been largely off‐label (Oswald and Sonenklar 2007) and often not directed toward core symptoms of the condition (Gibbs 2010). In a seminal clinical trial by King *et al. *(2009), citalopram hydrobromide, an SSRI that primarily targets 5‐HTT, was administered to 149 children with ASD aged 5–17 years for 12 weeks from six academic institutions. Instead of seeing significant improvement in symptoms and/or behavior, the citalopram hydrombromide group experienced adverse effects of the drug, including increased energy level and hyperactivity, insomnia, reduced concentration, impulsiveness, and stereotyped behaviors. Recently, a case report in which four children 6–12 years old with ASD were given low doses of the SSRI fluoxetine found improvement in attention‐deficit/hyperactivity disorder‐like symptoms, irritability and/or self‐injurious behavior (Lucchelli and Bertschy 2018). Previously, Hollander *et al. *(2012) found improvement in repetitive behaviors in adult ASD subjects who were administered fluoxetine while McDougle *et al. *(1996), in a clinical trial using the SSRI fluvoxamine, reported improvement in aggressive behavior in adult ASD patients. However, a Cochrane Review including 320 participants concluded there is no evidence for SSRIs as effective treatments for autism in children, but there is limited evidence for effectiveness in adults (Williams *et al.* 2013). These and other studies suggest that there are differential effects depending on the specific dose‐dependent SSRI administered, with citalopram being the least effective in ameliorating or modulating autism‐related behaviors.

Nevertheless, there is substantial evidence that 5‐HT systems are altered in ASD in both humans and in animal models (for review see: Muller *et al.* 2016). Many individuals with ASD and some of their family members have increased blood 5‐HT levels (Schain and Freedman 1961; Cook and Leventhal 1996; Lam *et al.* 2006; Gabriele *et al.* 2014) and genetic variation in 5‐HTT in some individuals with autism has been reported (Yonan *et al.* 2003; Devlin *et al.* 2005; Sutcliffe *et al.* 2005; Kistner‐Griffin *et al.* 2011). Positron emission tomography (PET) imaging studies have demonstrated abnormal 5‐HT synthesis in brain regions in autism (Chugani *et al.* 1997; Chugani *et al.* 2001; Chandana *et al.* 2005) and a polymorphism in the gene for the serotonin transporter (SLC6A4) has been associated with increased cerebral cortex grey matter volume in children with ASD (Wassink *et al.* 2007).

These early clinical, imaging, and genetic studies demonstrated 5‐HT alterations in the autism brain, supported the use of 5‐HT as a biomarker and suggested the potential for novel biomarkers to be found in different 5‐HT receptor subtypes, which led to several imaging studies that focused primarily on high‐functioning individuals with autism. A PET imaging study of a high‐functioning autism cohort (*n* = 20, 18–26 years of age) revealed decreased 5‐HTT binding in the anterior cingulate cortex (ACC), posterior cingulate cortex (PCC) and thalamus, which was associated with behavioral changes, including impairment of social cognition and repetitive/obsessive actions and interests (Nakamura *et al.* 2010). Makkoen *et al. *(2008) used single photon emission computed tomography on a small group of high‐functioning children and adolescents and found decreased 5‐HTT binding in the medial frontal lobe, especially in the adolescent cohort, whereas 5‐HTT binding in the midbrain and temporal lobe was normal. In another imaging study, Murphy *et al. *(2006) utilized the ligand ^123^I‐5‐I‐R91150 that shows selectivity for 5‐HT_2_ receptors and reported significant binding decreases associated with social communication deficits. Additionally, parents of children with ASD have been reported to have significantly decreased cortical 5‐HT_2_ receptor binding, with a negative correlation to their platelet 5‐HT levels using PET imaging (Goldberg *et al.* 2009).

In contrast to high‐functioning autism, there is a paucity of investigations that include idiopathic autism cases, making it difficult to generalize potential pharmacotherapies toward affected receptors across the autism spectrum. In one of the few studies of idiopathic autism, Azmitia *et al. *(2011) utilized immunocytochemistry in postmortem autism and neurotypical control cases 2–29 years to label serotonergic fibers in the forebrain cortical white matter and their projections into the temporal cortex. These investigators identified an increased number of 5‐HT axons in the autism group, leading to the conclusion that physicians should use caution in prescribing SSRIs to children. These authors also postulated that the increased number of 5‐HT axons could have profound effects on 5‐HT receptor subtypes, including the presynaptic 5‐HT_1A_ receptors and the abundant 5‐HT_2A_ receptors, thus affecting synaptogenesis and ultimately function.

However, there are very few studies analyzing 5‐HT receptor differences within the postmortem brain of individuals with autism. Our initial findings based on single concentration ligand binding studies in young adults with ASD provided strong evidence for alterations in brain 5‐HT systems in autism due to the high sensitivity of autoradiography methods, with reductions in density of 5‐HT_1A_ and 5‐HT_2A_ receptors in the PCC and fusiform gyrus (FG), but no change in binding to the transporter 5‐HTT (Oblak *et al.* 2013). This suggested that pharmacotherapies targeted toward affected 5‐HT receptor subtypes identified within different individuals may lead to improved therapeutic outcomes.

We thus aimed to expand on our initial single concentration ligand study (Oblak *et al.* 2013) by increasing the cohort size and comparing multiple concentrations of three 5‐HT ligands in three cortical regions within ASD cases that span a range of severity in diagnosis. In addition to the PCC and FG, we included the ACC, which had not been evaluated in the previous study. *Here, we perform multiple concentration saturation binding assays in a cohort of human postmortem brain tissue from individuals with idiopathic autism as compared to age‐matched neurotypical control brains*. This design allows for determination between a change in receptor density (*B*_max_) and/or a change in ligand affinity (*K*_D_), which might arise due to post­translational modification of receptors, receptor oligomerization, or association of receptors with modulatory proteins (Nichols and Nichols 2008). 5‐HT_1A_ and 5‐HT_2_ receptors, as well as the transporter 5‐HTT, were quantified in both superficial and deep laminar layers in the ACC, PCC and FG. This larger cohort allowed for separation of cases based on age (adults > 16 years and children/adolescents ≤ 16 years) to mimic clinical trials that found differential age‐related results with SSRI use.

In one of the largest postmortem autism studies to date, our results indicate alterations in the 5‐HT system within the adult ACC. The ACC is one of the most consistently reported abnormal cortical regions in behavioral, imaging, and pathological studies in autism (Bauman and Kemper 1985; Bailey *et al.* 1998; Simms *et al.* 2009; Forde *et al.* 2018) and plays fundamental roles in cognitive processes such as motivation, decision making, learning, cost‐benefit calculation, as well as conflict and error monitoring (Shackman *et al.* 2011; Holroyd and Yeung 2012; Rushworth *et al.* 2012; Shenhav *et al.* 2013; Ullsperger *et al.* 2014; Holroyd and McClure 2015; Verguts *et al.* 2015; Laubach *et al.* 2015; Kolling *et al.* 2016; Apps *et al.* 2016). An emerging role for the ACC in social cognition, particularly tracking the motivation of others (for review see: Apps *et al.* 2016), may be especially relevant in the context of ASD as it is thought that autistic individuals lack social motivation (Mundy and Neal 2000; Dawson *et al.* 2005; Schultz 2005; Chevallier *et al.* 2012). Therefore, the ACC may represent a vulnerable area in ASD, which has been shown to have relevance to ASD specific behaviors.

## Materials and methods

### Postmortem tissue

Human postmortem brain tissue was obtained from the University of Maryland Brain and Tissue Bank, a brain and tissue repository of the NIH Neurobiobank, and from the Harvard Brain and Tissue Resource Center of the Autism Tissue Program, which is now Autism BrainNet. Case demographics are provided in Table 1. Fresh frozen tissue blocks from the left hemisphere of three cortical regions including the ACC (*n* = 19 control, *n* = 18 autism), PCC (*n* = 18 control, *n* = 19 autism), and FG (*n* = 18 control, *n* = 14 autism) were sectioned coronally at 20 µm on a cryostat and kept frozen at −80˚C. There were no statistically significant differences in total case ages (*p* = 0.14) or postmortem interval (*p* = 0.23) between autism and control cases using a Welch’s *t*‐test. All autism cases had confirmed diagnoses through the autism diagnostic interview‐revised (ADI‐R) scores and/or received a clinical diagnosis of autism from a licensed psychiatrist. Ten autism cases had at least one seizure reported and seven had reported medications (Table 1).

**Table 1:** Postmortem brain donor case demographics.

Cases	Diagnosis	Age	PMI	Gender	Ethnicity	Cause of Death	Brain Bank‐ Seizure
**Adults >16**							
602	Control	27	15	M	Caucasian	Accident, multiple injuries	UMBTB
1026	Control	28	6	M	Caucasian	Congenital Heart Disease	ATP
1365	Control	28	17	M	Caucasian	Multiple injuries	ATP
4103	Control	43	23	M	Caucasian	Heart attack/disease	ATP
4104	Control	24	5	M	African American	Gun shot to the chest	ATP
4267	Control	26	20	M	African American	Accident	ATP
4268	Control	30	22	M	African American	Cardiomyopathy (heart attack/disease)	ATP
4271	Control	19	21	M	African American	Epiglottis/unknown	ATP
4272	Control	19	17	M	Caucasian	Accident	ATP
4275	Control	20	16	M	Caucasian	Accident	ATP
4345	Control	27	19	F	Caucasian	Respiratory failure	UMBTB
4364	Control	27	27	M	Unknown	Motor vehicle accident	ATP
4599	Control	23	18	M	African American	Cardiac arrthymia/anomalous coronary artery	UMBTB
4605	Control	29	17	M	African American	Commotio cordis	ATP
4916	Control	19	5	M	Caucasian	Accident, drowning	ATP
5321	Control	19	12	F	Unknown	Accident	ATP
5813	Control	20	24	M	African American	Atherosclerotic cardiovascular disease	UMBTB
5873	Control	28	23	M	African American	Unknown	ATP
6004	Control	36	18	F	African American	Unknown	ATP
1078	Autism	22	14	M	Caucasian	Congenital heart disease	ATP‐ Yes
1401	Autism	21	20	F	Caucasian	Sepsis	ATP
1484	Autism	19	15	M	English‐white	Burns	ATP
1664	Autism	20	15	M	Unknown	Drowning	ATP‐ Yes
2825^a^	Autism	19	9	M	Unknown	Cardiac arrest	ATP‐ Yes
3845	Autism	30	28	M	English‐white	Cancer	ATP
4099	Autism	19	3	M	Caucasian	Drowning	ATP
5027	Autism	37	26	M	African american	Obstruction of bowel due to adhesion	UMBTB‐ Yes
5574	Autism	23	14	M	African american	Pneumonia	UMBTB
5754	Autism	20	29	M	Uknown	Unknown	ATP
5771^b^	Autism	27	5	F	Caucasian	Undetermined	UMBTB‐ Epilepsy
5864^c^	Autism	20	63	M	Caucasian	Seizure disorder	UMBTB
6337	Autism	22	25	M	Caucasian	Aspiration	ATP
**Children/adolescents ≤16**							
1714	Control	12	22	M	African American	Cardiac arrthymia	UMBTB
3835	Control	9	8	F	African American	Unknown	UMBTB
4188	Control	16	13	M	African American	Gunshot	ATP
4274	Control	16	15	F	Unknown	Accident	ATP
4337	Control	8	16	M	African American	Blunt force neck injury	UMBTB
4670	Control	4	17	M	Caucasian	Commotio cordis	UMBTB
5170	Control	13	20	M	African American	Gunshot wound to chest	UMBTB
5242	Control	15	9	M	Caucasian	Cardiac arrhythmia	UMBTB
5334	Control	12	15	M	Hispanic	Hanging/ suicide	UMBTB
5376	Control	13	19	M	Caucasian	Hanging/suicide	UMBTB
5387	Control	12	13	M	Caucasian	Drowning	UMBTB
5391	Control	8	12	M	Caucasian	Drowning	UMBTB
5408	Control	6	16	M	African American	Drowning	UMBTB
5558	Control	5	19	M	Caucasian	Anomalous left coronary artery w/ complications	UMBTB
1469	Autism	5	42	M	English‐white	Unknown	ATP
2004	Autism	10	23	M	Asian	Drowning	UMBTB
2993	Autism	5	4	M	English‐white	Acute respiratory distress syndrome	ATP‐ Yes
3871	Autism	5	25	M	Caucasian‐Hispanic	Drowning	UMBTB‐ Yes
3924	Autism	16	9	F	Portuguese/Spanish	Epilepsy	UMBTB‐ Epilepsy
4334^d^	Autism	11	27	M	Hispanic	Acute hemorrhagic tracheobronchitis	UMBTB
4721	Autism	8	16	M	African american	Drowning	UMBTB
4849	Autism	7	20	M	African american	Drowning	UMBTB
4899^e^	Autism	14	9	M	Caucasian	Drowning	UMBTB
5144	Autism	7	3	M	Caucasian	Complications of cancer	UMBTB‐ 1 week
							before death
5302	Autism	16	20	M	Caucasian	Diabetic ketoacidosis	UMBTB
5308	Autism	4	21	M	Caucasian	Skull fractures	UMBTB‐ Disorder
5565^f^	Autism	12	22	M	African american	Seizure disorder, complications	UMBTB
5841^g^	Autism	12	15	M	Caucasian	Hanging	UMBTB

PMI, postmortem interval.

Known medications:

Tegretol, Phenobarbital, Theodor.

Ranitidine, Hydrocodone, Lamictal, Proferrin, Aranesp, Zonegran, Diovan, Microgestin.

Neurontin, Risperidone, Lorazepam, Oxcarbazepine.

Kepra, Lexapro, Biaxin.

Clonidine, Melatonin, Trileptal, Zoloft.

Kepra.

Daytrana, Vyvanse.

As this reasearch did not involve live human subjects, Institutional Review Board approval and informed consent was not necessary. However, the University of Maryland Brain and Tissue Bank is overseen by Institutional Review Board protocol number HM‐HP‐00042077. This study was not pre‐registered.

### Saturation ligand binding assays

Three tritiated [^3^H] ligands (Perkin Elmer, Boston, MA, USA) including ketanserin [NET7910 (2015)] (specific activity (SA) = 47.3 Ci/mmol), citalopram [NET1039 (2015)] (SA = 82.1 Ci/mmol), and 8‐OH‐DPAT [NET929 (2015)] (SA = 146 Ci/mmol) were utilized as outlined in Table 2. All tissues included in each assay were processed under the same conditions at seven different concentrations that spanned 0.51 to 139nM in order to obtain saturation binding curves for each ligand (Oblak *et al.* 2011). Two thawed 20 µm sections from each case were used to quantify total binding at each concentration of ligand and one section from each case was exposed to the ligand as well as 100 µM of a displacer (imipramine hydrochloride AAJ6372306, Fisher Scientific, Waltham, MA, USA (2016); ritanserin 1955, Tocris Biosciences, Bristol, UK (2016) and serotonin hydrochloride 3547, Tocris Biosciences (2016)) to determine non‐specific binding (Table 2), which was subtracted from the total binding to yield specific binding of the ligand to the respective receptor subtype. All tissues went through a pre‐incubation in buffer without ligand (5‐HT_2_: 50 mM Tris‐HCl (pH 7.4), 4 mM CaCl2, 0.1% ascorbate; 5‐HTT: 50 mM Tris‐HCl (pH 7.4), 120mM NaCl, 5mM KCl; 5‐HT_1A_: 170 mM Tris‐HCl (pH 7.6), 4 mM CaCl2, 0.01% ascorbate) for 30 min before being exposed to the ligand and were then rinsed three times for 5 min in buffer followed by one dip in distilled water and allowed to air‐dry overnight. Slides were placed in X‐ray cassettes with [^3^H]‐sensitive hyperfilm [Kodak Biomax MR film Z350389; Sigma‐Aldrich, St. Louis, MO, USA (2016)] and a [^3^H] standard [Tritium standards ART0123; American Radiolabeled Chemicals, St. Louis, MO, USA (2015)] then exposed for 3–26 weeks. Films were processed in a darkroom by developing for 3 min in developer [Kodak D19 74200; Electron Microscopy Sciences, Hatfield, PA, USA (2015)], fixed [Kodak Rapidfix 74312; Electron Microscopy Sciences (2015)] for 4 min at 23°Cand washed with a stream of water for at least 1 h and air‐dried.

**Table 2:** Multiple concentration binding conditions for serotonin receptor tritiated ligand binding in the ACC, PCC, and FG

Receptor	Ligand	*K*_D_	Concentrations (nM)	Incubation	Displacer	Exposure (weeks)
5‐HT2	[^3^H] Ketanserin	0.5	0.53, 1.0, 2.8, 9.4, 32.5, 89.6, 116.0	30 min	Imipramine	3–24
5‐HTT	[^3^H] Citalopram	3.0	0.51, 1.4, 3.6, 7.8, 30.6, 89.2, 127.0	1 h	Ritanserin	5–24
5‐HT_1A_	[^3^H] 8‐OH‐DPAT	2.0	0.53, 1.5, 2.9, 10.7, 29.1, 91.0, 138.9	1 h	5‐HT Hydrochloride	4–26

5‐HT, 5‐hydroxytryptamine (serotonin); 5‐HTT, serotonin transporter; ACC, anterior cingulate cortex; FG, fusiform gyrus; PCC, posterior cingulate cortex.

### Data analysis

Autoradiographs of the tissue sections exposed to film were digitized with a QICAM digital camera (QImaging, Surrey, BC, Canada) and analyzed using MCID Core 7.1 Elite Image analysis system software (InterFocus Imaging Ltd, UK). Calibration of each film with its own [^3^H] standard allowed for comparisons across films by choosing one representative standard curve and normalizing all other standard curves and images to it within the MCID software (Linton, UK). The standard curves allowed for conversion from optical density to nanocuries per milligram (mg).

**Figure 1:**
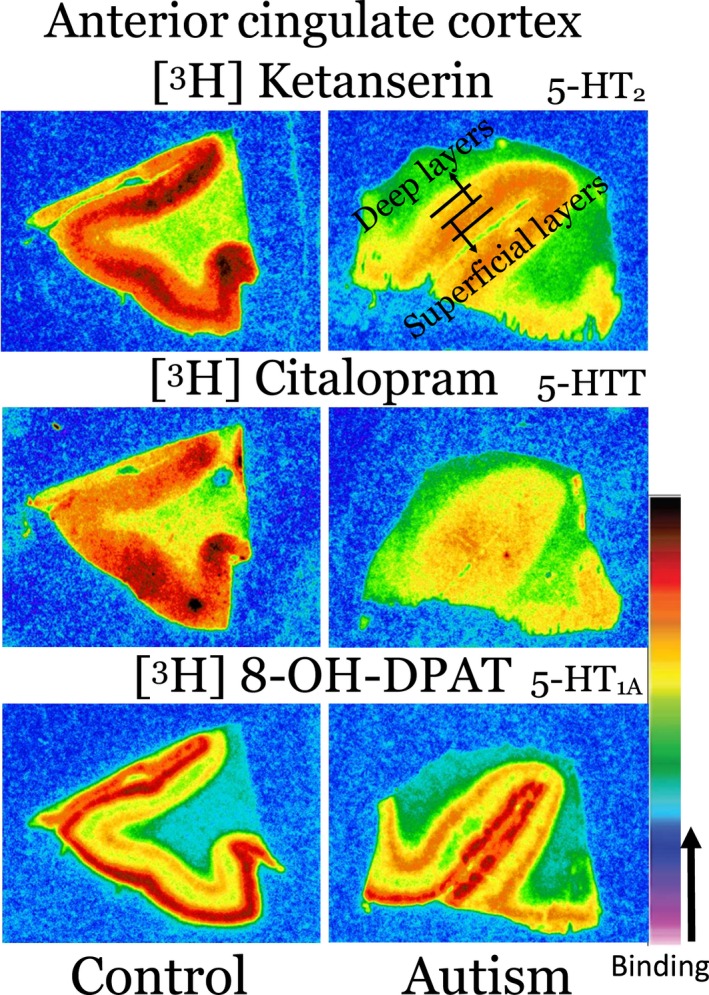
Example pseudo‐colored ligand binding images from the ACC. Red represents higher laminar binding. Cortical layers were separated into superficial and deep as shown in the top right image. The same adult case, one control (case 4599, age 23) and one autism (case 5864, age 20) is given for each ligand at 9nM. [^3^H]‐ ketanserin and [^3^H]‐citalopram have similar binding distribution in the deep and superficial layers, whereas [^3^H]‐8‐OH‐DPAT has higher binding in the superficial layers. Note the lower binding in the autism case for 5‐HT_2_ and 5‐HTT, whereas the 5HT_1A_ has the same level of binding as the control. ACC, anterior cingulate cortex; 5‐HT, 5‐hydroxytryptamine (serotonin).

Binding in the superficial layers (I–IV) and deep layers (V–VI) of each cortical region (Fig. 1) was sampled with the ribbon tool running along the length of the observable region of interest then converted to femtomoles per mg of protein based on the specific activity of each ligand. The ACC, a limbic cortical area, lacks layer IV, thus superficial sampling in the ACC includes layers I–III. The experimenter was blinded to the diagnosis of the cases during sampling. After background subtraction, these total binding values were plotted against the concentration of ligand and fitted with non‐linear least squares regression to generate saturation binding curves in GraphPad prism 6 to estimate the number of receptors (*B*_max_) and binding affinity (*K*_D_) of each ligand for each case.

### Statistical analyses

Equality of variance was calculated with an *F*‐test, and since many of the autism ligand groups compared to the control groups did not show equality of variance, Welch’s *t*‐tests were performed to determine differences in the number of estimated total receptors (*B*_max_). Since the statistical distribution of fitted *K*_D_ values is log‐normal (Delean *et al.* 1982), averaging, calculation of standard errors, and significance testing were carried out on the negative logarithm of *K*_D_ (pK_D _= −log*K*_D_) (Oblak *et al.* 2009). Student’s *t*‐tests were conducted to compare binding affinity for each ligand in each brain region (Table 3). An assessment of normality was conducted using the Shapiro–Wilk test and both control and autism *B*_max_ and *K*_D_ values followed a normal distribution. Regression analyses were carried out in Microsoft Excel (2013) and fitted with trendlines to obtain *R* ^2^ values. Student’s *t*‐tests were utilized to compare the slope and intercept (elevation) of the trendlines between autism and control groups. As we used all available tissue obtained from the respective brain banks, a power analysis was not carried out and no tests for outliers were conducted.

**Table 3:** *B*_max_ and *K*_D_ for each individual ACC case in both deep and superficial layers

	**Anterior Cingulate Cortex**											
	**Deep Layers**						**Superficial Layers**					
**CASES**	[3H] Ketanserin		[3H] Citalopram		[3H] 8OH‐DPAT		[3H] Ketanserin		[3H] Citalopram		[3H] 8OH‐DPAT	
**Adults**	Bmax	pKD	Bmax	pKD	Bmax	pKD	Bmax	pKD	Bmax	pKD	Bmax	pKD
4103	1811	7.50	735.3	6.78	197.6	7.24	1706	7.55	531.2	6.97	208.5	7.99
4104	1205	7.81			218.2	7.22	1209	7.79			242.6	7.63
4267	2088	7.41	211.3	7.53	306.0	6.82	2159	7.38	216.8	7.55	243.2	7.60
4268	4224	6.85	846.5	6.68	294.0	6.70	3489	6.99	778.0	6.75	181.0	7.78
4271	590.2	7.76	596.5	6.31	263.6	7.02	606.9	7.78	596.0	6.33	308.2	7.34
4272	2277	7.44	479.2	7.03	227.8	7.10	2295	7.45	805.7	6.72	265.6	7.68
4275	1025	7.85	201.8	7.24	89.2	7.85	1062	7.81	202.6	7.26	125.7	8.12
4345	4241	6.83	289.1	7.03	190.2	7.47	3242	7.05	256.8	7.13	266.7	7.92
4364	2130	7.37	166.9	7.63	163.1	7.14	2137	7.38	168.5	7.63	169.1	8.03
4599	2676	7.32	488.9	6.96	185.9	7.35	2388	7.40	484.2	6.97	235.2	7.75
5813	1219	7.76	170.2	7.17	676.3	6.62	1261	7.75	167.1	7.22	210.3	8.03
**Mean**	**2135**	**7.45**	**418.6**	**7.04**	**255.6**	**7.14**	**1960**	**7.48**	**420.7**	**7.05**	**223.3**	**7.81**
**±SEM**	**364**	**0.11**	**78.6**	**0.12**	**45.9**	**0.11**	**271**	**0.09**	**79.4**	**0.12**	**15.5**	**0.07**
1078	767.8	7.35	249.3	6.75	327.4	6.71	803.1	7.30	176.5	7.03	407.8	6.69
1401	1158	7.73	204.1	6.90	183.0	7.14	1171	7.75	201.2	6.93	355.9	7.12
1484	1466	7.66	170.0	7.39	319.4	6.95	1558	7.60	172.8	7.40	322.7	7.15
1664	745.6	7.75	202.7	6.80	304.1	7.06	764.3	7.72	272.4	6.59	339.5	7.32
3845	1601	7.48	186.9	7.72	80.1	7.83	1625	7.46	193.7	7.71	117.4	8.22
5574	1474	7.80	302.8	7.36	520.5	6.68	1567	7.80	311.4	7.39	288.2	7.41
5754	1265	7.80	274.1	7.23	630.2	6.27	1299	7.74	278.6	7.24	173.5	7.96
5771	1573	7.71	303.4	7.03	518.0	6.79	1690	7.69	318.3	7.01	289.9	7.72
5864	1295	7.47	237.6	7.41	297.1	7.11	1412	7.41	222.5	7.57	249.1	7.60
**Mean**	**1261**	**7.64**	**236.8**	**7.18**	**353.31**	**6.95**	**1321**	**7.61**	**238.6**	**7.21**	**282.7**	**7.47**
**±SEM**	**107**	**0.05**	**16.5**	**0.11**	**58.1**	**0.14**	**115**	**0.06**	**19.1**	**0.12**	**30.3**	**0.16**
**T‐test**	*0.0402	**0.1502**	*0.04752	**0.4105**	**0.2055**	**0.2857**	*0.0489	**0.2687**	*0.0497	**0.3769**	**0.1068**	*0.0479
**Children**												
3835	2312	7.27	404.8	6.88	101.4	7.88	2129	7.38	364.8	6.95	155.1	7.88
4188	1089	7.73	328.8	7.32	317.0	7.06	1168	7.67	276.4	7.44	330.9	7.41
4274	827.9	8.00	186.8	7.36	288.6	7.10	852.3	7.99	173.1	7.45	275.6	7.20
5242	1597	7.51	287.9	7.28	144.7	7.51	1464	7.62	295.1	7.25	196.2	7.95
5334	1760	7.46	296.4	7.21	237.5	7.22	1585	7.54	294.9	7.23	211.7	7.86
5376	2866	6.92	374.9	7.05	315.7	7.03	2551	6.98	358.3	7.08	223.6	7.80
5387	2194	7.02	842.1	6.29	395.3	6.90	2188	7.02	732.6	6.36	257.7	7.82
5391	1848	7.55	314.5	6.81	160.4	7.68	1751	7.60	413.9	6.65	229.3	7.93
**Mean**	**1812**	**7.43**	**379.5**	**7.03**	**245.1**	**7.30**	**1711**	**7.48**	**363.6**	**7.05**	**235.0**	**7.73**
**±SEM**	**233**	**0.13**	**69.9**	**0.13**	**36.0**	**0.12**	**199**	**0.12**	**58.6**	**0.14**	**18.9**	**0.10**
1469	695.3	7.78	123.4	7.33	806.3	6.10	725.3	7.76	124.9	7.30	323.0	6.59
2993	3435	6.63	82.3	7.28	80.2	7.77	3777	6.57	85.7	7.26	93.9	8.15
3871	2314	7.37	144.0	7.32	315.5	6.69	2320	7.39	148.5	7.29	231.7	7.64
3924	1344	7.83	169.2	7.49	106.6	7.99	1399	7.81	176.2	7.48	160.2	8.20
4334	2789	7.18	662.4	6.56	334.1	6.95	2793	7.19	568.2	6.66	248.1	7.67
4721	2369	7.30	228.7	7.03	158.2	7.48	2104	7.41	224.7	7.05	193.6	7.90
5302	1700	7.72	381.9	7.18	592.3	6.60	1711	7.73	375.0	7.25	216.6	7.53
5565	2035	7.51	356.8	6.95	219.1	7.44	1965	7.54	383.7	6.89	257.7	7.63
5841	1720	7.57	183.8	7.19	145.0	7.66	1713	7.61	245.6	6.93	199.6	8.07
**Mean**	**2045**	**7.43**	**259.16**	**7.15**	**306.37**	**7.19**	**2056.4**	**7.44**	**259.17**	**7.12**	**213.82**	**7.71**
**±SEM**	**269**	**0.12**	**60.7**	**0.09**	**81.6**	**0.21**	**289**	**0.13**	**51.8**	**0.09**	**21.5**	**0.16**
**T‐test**	**0.5229**	**0.9940**	**0.2140**	**0.4462**	**0.5062**	**0.6723**	**0.3423**	**0.8641**	**0.2020**	**0.6483**	**0.4701**	**0.9038**
**T‐test all cases control vs autism**												
	**0.2339**	**0.3763**	*0.0174	**0.2488**	**0.1807**	**0.3654**	**0.5145**	**0.6474**	*0.0162	**0.3228**	**0.3930**	**0.1479**

ACC, anterior cingulate cortex.

*T*‐tests were performed as described in methods to compare autism (orange) to controls (black) within groups of adults only, children only, and total cases. Asterisks mark significantly different group means.

## Results

Each of the three cortical regions, ACC, PCC, and FG were included in all three ligands; [^3^H]‐ketanserin (5‐HT_2_), [^3^H]‐citalopram (5‐HTT), and [^3^H]‐8‐OH‐DPAT (5‐HT_1A_). The superficial (Lamina I–III in ACC; I–V in PCC and FG) and deep layers (Lamina V–VI) were approximately delineated from adjacent Nissl stained sections and autoradiographs as shown in Fig. 1. Anatomical levels were matched as much as possible based on whole tissue blocks provided by the brain banks. The receptor distribution pattern based on the ligand binding was mostly homogeneous with [^3^H]‐ketanserin and [^3^H]‐citalopram across layers, whereas [^3^H]‐8‐OH‐DPAT had high binding in the superficial layers of the cortex as compared to the deep layers at most ligand concentrations, which saturated at a similar level with higher concentrations of ligand. The autism ACC case shown in Fig. 1 had similar binding to the control case for 5‐HT_1A_, but had lower binding for both 5‐HT_2_ and 5‐HTT indicating receptor‐specific alterations in the serotonergic system in some autism cases within the ACC.

**Figure 2:**
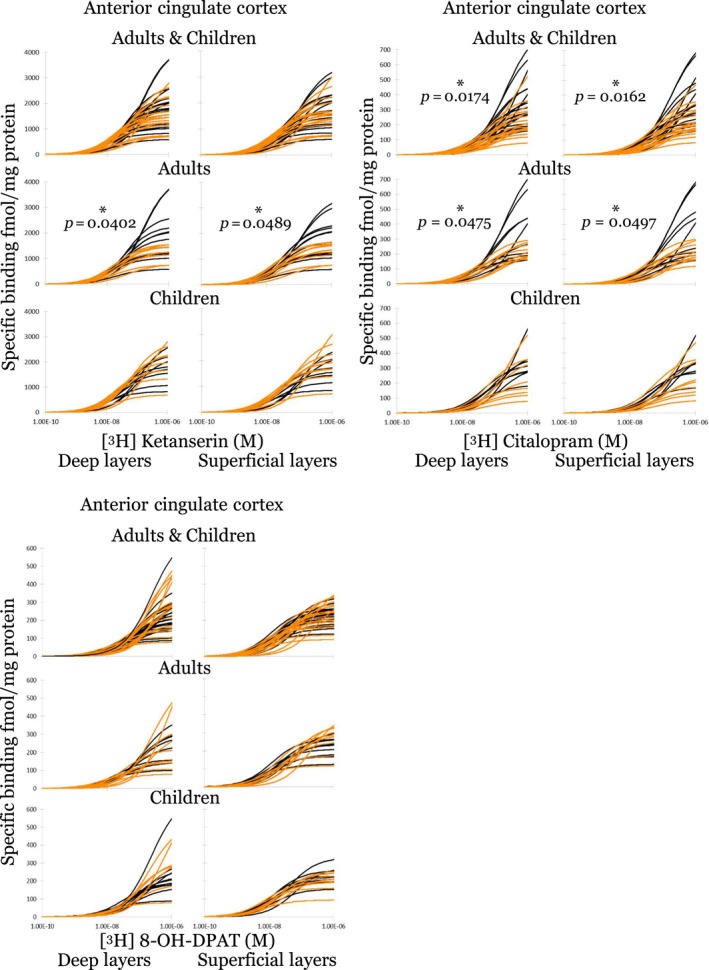
Individual saturation binding curves for all cases in the ACC. Individual curves each include seven concentrations of ligand. Autism (orange) cases had significantly decreased binding in both superficial and deep layers for 5‐HTT ([^3^H]‐citalopram) compared to control cases (black) that is driven by a lower *B*_max_ in adult autism cases. Similarly, 5‐HT_2_ ([^3^H]‐ketanserin) receptor binding (*B*_max_) was decreased in adult cases (*n* = 11 control and 9 autism), but not in children (*n* = 8 control and 9 autism). 5‐HT_1A_ [^3^H]‐8‐OH‐DPAT) binding was unchanged for each of the three groups; total autism and control cases, adults only and children only. *n* = individual postmortem cases. ACC, anterior cingulate cortex; 5‐HT, 5‐hydroxytryptamine (serotonin).

These alterations are readily observed when comparing saturation binding curves in the ACC for control cases versus autism cases (Fig. 2). There were no group differences when comparing the *B*_max_ or *K*_D_ of all autism and control cases for 5‐HT_1A_ and 5‐HT_2_, however, a decrease in *B*_max_ for 5‐HTT binding was significant for the autism group (Table 3). Intriguingly, when separating total control and autism cases into adults (> 16 years) and children (≤16 years) it appears that the adult cases are mostly driving the group decrease in 5‐HTT binding. Furthermore, separating analysis of children and adults for 5‐HT_2_ binding revealed a statistically significant decrease in *B*_max_ for adult autism cases that was not apparent when comparing the autism and control group as a whole. This indicates a similar receptor number in the ACC when comparing controls and children with autism, but a tendency for autism cases to have lower binding levels of 5‐HTT and 5‐HT_2_ than controls in adulthood.

**Figure 3:**
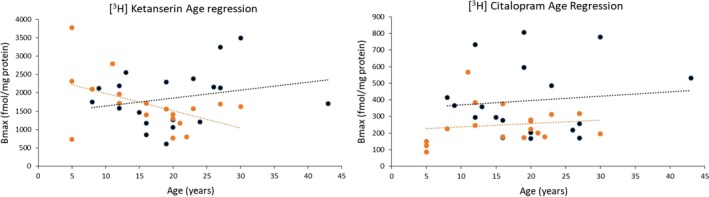
Age regression analysis. Receptor expression (*B*_max_) plotted against age for both [^3^H]‐ketanserin (5‐HT_2_) and [^3^H]‐citalopram (5‐HTT) in the ACC. [^3^H]‐ketanserin had a decreasing slope in the autism group (*n* = 18) that was significantly different (*p* = 0.032) from controls (*n* = 19). [^3^H]‐citalopram had no difference in slope (*p* = 0.94), but had a significantly lower intercept (elevation) compared to controls (*p* = 0.025). *n* = individual postmortem cases. ACC, anterior cingulate cortex; 5‐HT, 5‐hydroxytryptamine (serotonin).

While the separation of children and adults mirrors clinical studies, the cutoff age of 16 years isn’t necessarily biologically relevant, as development occurs over a continuous time course. Therefore, further analyses were carried out to more clearly define the relationship between age and 5‐HT receptor expression. Plotting *B*_max_ values against age (Fig. 3) reveal similar slopes for 5‐HTT density (*p* = 0.94), but an intercept (elevation) that is significantly lower in the autism group across ages (*p* = 0.025). 5‐HT₂ density increases with age in control cases, whereas in autism there is a decrease with age and significantly different slopes between regression lines (*p* = 0.032).

The results from the three ligands and three cortical regions also indicate that disruptions in the serotonergic system are region specific. There were no differences in *B*_max_ or *K*_D_ for any ligand in the PCC or FG (see Figures S1, S2; Tables S1, S2). There were not enough female cases in the cohort to analyze differences between gender; however, the two female cases (1401 and 5771) in the adult autism group of the ACC had *B*_max_ values that fell in the normal range of controls but higher values of the autism group, suggesting the need to analyze a larger cohort to determine if this decrease in serotonin receptors is gender specific for males.

Since some of the autism cases had a history of at least one seizure, Welch’s *t*‐tests were conducted to compare the *B*_max_ means for each ligand between autism cases with a seizure history and without. There were no statistical differences in group means in the ACC autism cases for neither [^3^H] ketanserin (seizure mean = 2026 ± 376.6, no seizure mean = 1520 ± 177.0, *p* = 0.26), [^3^H] citalopram (seizure mean = 187.3 ± 31.91, no seizure mean = 279.7 ± 34.44, *p* = 0.07) nor [^3^H] 8‐OH‐DPAT (seizure mean = 224.6 ± 34.53, no seizure mean = 260.1 ± 24.62, *p* = 0.42). For the two ligands showing significant differences in autism, *B*_max_ means were not different based on source of the tissue (ATP vs. UMBTB Table 1), as [^3^H] ketanserin *p* = 0.09 and [^3^H] citalopram *p* = 0.53. There was also no correlation of *B*_max_ values to postmortem interval (Figure S3).

**Figure 4:**
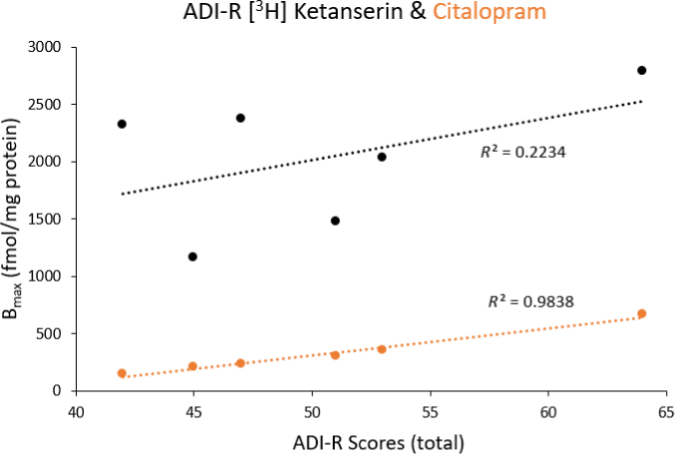
Symptom severity compared to receptor expression. Receptor expression (*B*_max_) plotted against autism diagnostic interview‐revised (ADI‐R) scores (*n* = 6) with *R* ^2^ values given. Higher ADI‐R scores represent increased severity of autism symptoms. *n* = individual postmortem autism cases.

Although the number of autism cases with ADI‐R scores available was limited for tissue obtained from the ACC, when plotting against *B*_max_ values (Fig. 4) a significant relationship between expression and symptom severity was observed for 5‐HTT (*R* ^2 ^= 0.9839), but not for 5‐HT₂ expression (*R* ^2 ^= 0.2234).

## Discussion

Given the longstanding implications of 5‐HT dysfunction in ASD and common use of SSRIs to ameliorate adverse behaviors, it is important to determine which receptor subtypes may contribute to ASD symptoms and thus be amenable to pharmacological intervention within the central nervous system. In the present study, the ASD cohort from the PCC and FG displayed normal density and ligand affinity for 5‐HT_1A_, 5‐HT_2_, and 5‐HTT. However, in the ACC, while the 5‐HTT assay showed significant density decreases within the entire autism group (adults and children), this difference was most prominent in the adult autism cases, since the group of children mean 5‐HTT density remained unchanged. However, age regression analysis showed a consistent reduction in 5‐HTT across ages, as indicated by a lower elevation of the regression line. Whole group 5‐HT_2_ density was not altered in autism, however, when analyzing children and adults separately, the adult ACC autism cases again show significantly decreased density of 5‐HT_2_ receptors. Furthermore, 5‐HT_2_ receptor expression increased with age in controls, but decreased with age in autism. This demonstrates age‐dependent differences in 5‐HT within the ACC in a subset of ASD individuals. These findings are particularly intriguing in light of the clinical efficacy of SSRI use. SSRI drugs are commonly administered to individuals with autism, as up to 80% of children with ASD experience clinically significant anxiety (Muris *et al.* 1998; Leyfer *et al.* 2006; Simonoff *et al.* 2008). Nadeau *et al. *(2011) concluded, in a review of treatment studies, that cognitive‐behavior therapies may be more helpful than SSRI administration in youth with ASD. As reaffirmed in the 2013 Cochrane Review (Williams *et al.* 2013), there is no conclusive evidence that children benefit from SSRIs, but have been shown in major clinical trials to have significant adverse outcomes (e.g. King *et al.* 2009) in contrast to some adults with autism that do benefit from SSRIs. Our data parallels this information in that only a subset of adults with autism (> 16 years of age) have decreased density of 5‐HT_2_ and 5‐HTT in the ACC, but children appear to have normal levels in all three cortical regions evaluated. It remains to be explored whether there are similar 5‐HT receptor deficits in other brain regions in autism or whether the ACC is a more susceptible area than other cortical brain regions.

A possible explanation for inconsistent results within the literature regarding the 5‐HT system in ASD is the heterogeneity inherent to the condition amidst small sample sizes. For example, genetic polymorphisms in the 5‐HT transporter have been implicated in ASD (Yonan *et al.* 2003; Devlin *et al.* 2005; Sutcliffe *et al.* 2005; Kistner‐Griffin *et al.* 2011; Najjar *et al.* 2015), however, it is only a subset of individuals, presuming the majority of ASD cases do not show genetic differences in 5‐HTT. Furthermore, most imaging studies involving ASD, as discussed in the introduction, focus on high‐functioning individuals that may not be representative of the entire autism spectrum. The data presented here show mean decreases in receptor density in superficial and deep layers of the ACC in the adult autism groups, but several autism cases fall within the normal control range, indicating that not every autism case shows differences in specific 5‐HT receptor subtypes in the examined areas. Furthermore, while hyperserotonemia is considered the best replicated biomarker in autism, it still represents less than half of the ASD population (Gabriele *et al.* 2014). For these reasons, treatments for ASD should be tailored to the individual. As tests for peripheral hyperserotonemia and genetic variants become more widely available, future studies evaluating the efficacy of SSRIs in autism should divide individuals into subgroups based on evidence of 5‐HT disruption. As demonstrated by the data presented and by clinical SSRI outcomes, age of the individual may be a key factor for positive clinical outcomes, however, not all studies divide cohorts by age. For example, in a 6‐week open label trial with 44 participants with autism, two increasing daily doses of the SSRI escitalopram resulted in improvement on insistance of sameness and irritability symptoms irrespective of whether individuals had polymorphisms of 5‐HTT (SLC6A4) or of the 5‐HT2A receptor (HTR2A). However, differences in age groups were not reported despite subjects in the study falling between 5 and 44 years old (Najjar *et al.* 2015). Interestingly, symptom severity was correlated with 5‐HTT expression, but not with 5‐HT_2_ receptor expression. Higher ADI‐R scores represent increased symptom severity, which was correlated with higher 5‐HTT expression in a subset of autism cases in which ADI‐R scores were available. It is not clear why higher expression would be correlated with symptom severity, as control cases have higher expression. However, the expression of 5‐HTT is dependent on a diverse set of mechanisms (Daws and Gould 2011). As 5‐HTT is the uptake site of 5‐HT, its regulation is dependent on 5‐HT expression. If there is an increase in brain serotonin (central hyperserotonemia), a downregulation of 5‐HTT would help to offset its effects and if individuals were unable to downregulate 5‐HTT (due to a polymorphism or other reason), that heightened 5‐HT within the brain could be associated with more severe autistic symptoms. Further research is needed to clarify the development of the serotonergic system in autism, particularly in how receptors respond to altered levels of 5‐HT during development of the brain. The results of the present study also suggest that 5‐HT dysfunction may be secondary to other mechanisms or emerge as a compensation pattern within disrupted circuits. As suggested by our data, if the 5‐HTT reductions begin in childhood, it is possible that the reductions in 5‐HT_2_ in adults are occuring in response to this initial deficit. Further studies that evaluate changes in 5‐HT in ASD with age will be needed to determine whether children that have normal levels of 5‐HT receptors at a young age present with deficits later in life or whether these individuals had 5‐HT alterations throughout development. Since there were no differences in ligand binding affinity across each cortical region and ligand, it is unlikely our cohort had significant genetic risk in relationship to 5‐HT_1A,_ 5‐HT_2_, or 5‐HTT, which would again indicate a compensation pattern of 5‐HT alterations instead of a primary 5‐HT causative factor.

As individuals with ASD are commonly prescribed SSRIs at a young age, the need for informed use of pharmacological interventions is warranted. Our data adds to the growing literature that caution should be taken toward SSRIs in children under the age of 16 and that the 5‐HT system can be altered within distinct cortical regions and receptor subtypes.

### The ACC has consistent neurochemical, cytoarchitectural, and connectivity differences in adults with idiopathic autism

An intriguing result of the present study is the selective vulnerability of the ACC versus the PCC or FG due to changes in binding of 5‐HT_2_ receptors and 5‐HTT only in the ACC. Previous studies identified cytoarchitectual changes, including increased packing density, in ACC subregions and increased neurons in the white matter just below layer VI in about half of the adult autism postmortem cases in the study (Simms *et al.* 2009). A multimodal imaging study found that the ACC cytoarchitectural changes related to symptom severity in individuals with neurodevelopmental conditions including autism (Forde *et al.* 2018). In a recent landmark postmortem study of ACC connectivity, Zikopoulos *et al. *(2018) demonstrated an increased proportion of thin axons in the white matter just below the ACC from childhood through adulthood, which was indicative of hyper‐connectivity to short range neighboring cortices and a decreased proportion of thick diameter axons below the ACC, representing hypo‐connectivity of long range cortical connectivity.

Neurochemical differences in the autism ACC have been reported by Oblak *et al. *(2009, 2010). These authors reported significant decreases in GABA‐ARs and GABA‐BRs in the ACC in adult postmortem autism cases in superficial (I–III) and deep layers (V–VI). A recent proton magnetic resonance spectroscopic study by Ito *et al. *(2017) reported hypoGABAergic alterations in children with autism, but normal glutamate markers suggesting that the ACC may contribute to excitatory/inhibitory imbalance in subjects with idiopathic autism.

Using cognitive measures, behavioral analysis, and functional magnetic resonance imaging tasks in adults with idiopathic autism, Balsters *et al. *(2017) concluded that the ACC is a critical component of social behavior and that socially‐specific prediction errors occurred in the autism group. These authors also concluded that connectivity between the ventral medial prefrontal cortex and the ACC may represent a neural substrate underlying the social deficits. In fact, ACC activity can be monitored via 5‐HT_2_ receptors in rat pups as early as P10‐P12 and is modulated by maternal contact, which increases local field potentials in low frequency bands (Courtiol *et al.* 2018).

Collectively, these and other studies demonstrate that the ACC is a critical node for a variety of high‐order functions in the neurotypical brain. In autism, there are both structural changes in cortical lamination in the ACC in early development and through adulthood, as well as neurochemical changes in critical neurotransmitter systems, which includes GABA and serotonin as demonstrated by significant alterations in receptor densities across superficial‐deep lamina. This suggests that modulation of both input and output of the principal pyramidal neurons of the ACC may be impacted, potentially resulting in altered short and long‐range connectivity differences and affecting a wide range of autism‐related behaviors.

## Summary and conclusions

Some SSRIs administered to individuals with autism reported successful treatment of various symptoms, but citalopram hydrobromide had variable results that included harmful effects when administered to children. Previous studies have reported more favorable results when citalopram was administered to autistic individuals with specific polymorphisms. SSRIs mainly target the serotonin transporter and its blocking action allows 5‐HT to persist at the synapse. SSRIs are drugs that typically also affect other 5‐HT receptors, as well as dopamine and other receptor types. Multiple concentration ligand binding autoradiography is still a very useful tool to quantify the number (*B*_max_) and affinity (*K*_D_) of a variety of receptor types and was applied to this study investigating whether 5‐HTT and other major subtypes of 5‐HT receptors (5‐HT_2_ and 5‐HT_1A_) had significant changes in children and/or adult autism cohorts as compared to neurotypically developing individuals. Receptor density (*B*_max_) changes were seen in ^3^[H]‐citalopram labeled 5‐HTT and in ^3^[H]‐ketanserin labeled 5‐HT_2_ receptors in the adult autism group in the ACC only, but not in children. Furthermore, there were no changes in ^3^[H]‐8‐OH‐DPAT labeled 5‐HT_1A_ receptors in any of the groups or cortices. This study therefore provides further evidence that the ACC is a highly vulnerable region in autism, with changes in a number of receptor types that may contribute to the excitatory/inhibitory imbalance in the autism brain via disruptions in its cytoarchitecture and connectivity with other local and distant cortices. This could potentially have profound effects within cortical networks that, in part, underlie autism‐related behaviors.

## Supporting information


**Figure S1**. Individual saturation binding curves for all cases in the PCC.
**Figure S2**. Individual saturation binding curves for all cases in the FG.
**Figure S3**. Postmortem interval (PMI) versus receptor binding.
**Table S1**. *B*_max_ and *K*_D_ for each individual PCC case in both deep and superficial layers.
**Table S2**. *B*_max_ and *K*_D_ for each individual FG case in both deep and superficial layers.
