# Supplementary material for: Differential serotonin transporter (5‐HTT) and 5‐HT2 receptor density in limbic and neocortical areas of adults and children with autism spectrum disorders: implications for selective serotonin reuptake inhibitor efficacy
Source: J Neurochem. 2019 Oct 27;151(5):642–55. doi: 10.1111/jnc.14832 (PMC6900089; doi:10.1111/jnc.14832)
Supplement: Supplementary file 1 — Figure S1. Individual saturation binding curves for all cases in the PCC. Figure S2. Individual saturation binding curves for all cases in the FG. Figure S3. Postmortem interval (PMI) versus receptor binding. Table S1. B max and K D for each individual PCC case in both deep and superficial layers. Table S2. B max and K D for each individual FG case in both deep and superficial layers. [file JNC-151-642-s001.docx]

**Differential serotonin transporter (5-HTT) and 5-HT2 receptor density in limbic and neocortical areas of adults and children with ASD: implications for SSRI efficacy**

**Cheryl Brandenburg1 and Gene J. Blatt1**

**Program on Neuroscience1, Hussman Institute for Autism, Baltimore, MD 21201**

**Supplementary Information**

Posterior Cingulate and Fusiform Gyrus in autism – normal 5-HT markers despite previouly reported

neurochemical changes in structural/functional studies

Although our initial single concentration study in the PCC and FG showed significant changes in a number of 5-HT receptor subtypes (Oblak *et al.* 2013), the more comprehensive seven concentration saturation study differed from Oblak’s results, which included a smaller cohort of young adult autism cases. It must be noted, however, that the later study contained different autism and control cases than the earlier study. Therefore, it is likely that there are significant differences in 5-HT subtypes in the PCC and FG in some autism cases, but not as extensive as that seen in the ACC. It was somewhat surprising that the present results did not show significant changes in any of the three 5-HT receptor subtypes given that previous multiple concentration binding studies did demonstrate changes in GABA-ARs and GABA- BRs in the PCC and FG (Oblak *et al.* 2010, 2011a). However, in an earlier study, these authors reported normal neuron and interneuron density in both the PCC and FG, but did find cytoarchitectural alterations in the PCC of adult autism cases (Oblak *et al.* 2011b). The FG findings were in contrast to the van Kooten *et al.* (2008) postmortem study that reported cytoarchitectural abnormalities in the FG of autism cases, including smaller neurons and increased density of neurons. In sum, despite structural findings in the PCC and FG, the ACC stands out among the three cortices in having 5-HTR abnormalities in addition to the inhibitory GABA-A and GABA-B receptor differences in Bmax when performing comprehensive seven concentration ligand binding analyses. All three cortical areas have links to autism. While limbic ACC functions are varied, as described above, the limbic PCC is part of the default network (Greicius *et al.* 2003;

Buckner *et al.* 2008; Weng *et al.* 2010a) and has been reported to be affected in autism ([Kennedy & Courchesne 2008](https://www-ncbi-nlm-nih-gov.ezproxy.bu.edu/pmc/articles/PMC3110607/#R50)). The neocortical FG has a role in social cognition (e.g. Schultz *et al.* 2003) and is part of networks that are involved in facial processing, i.e., the fusiform face area (FFA; part of the FG), in which a number of studies of object recognition and/or face processing have shown differences, especially in high-functioning individuals with autism via functional imaging studies (e.g. Kanwisher *et al.* 1997 (review); [Pierce *et al.* 2001](https://www-ncbi-nlm-nih-gov.ezproxy.bu.edu/pmc/articles/PMC3110607/#R76); *W*eng *et al.* 2010b). Processing the faces of strangers has more pronounced differences than that of familiar faces (Pierce & Redclay 2008).


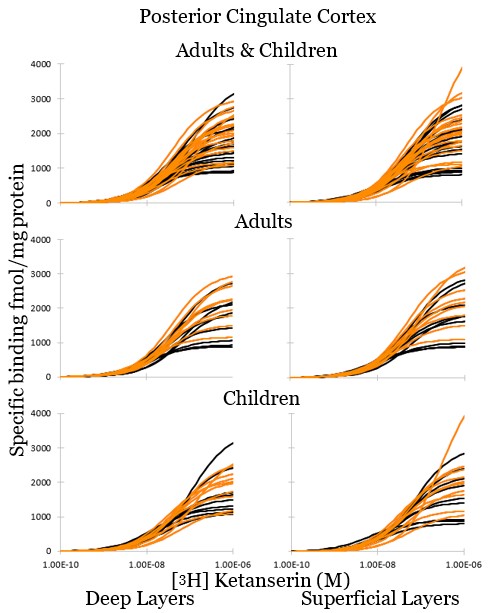


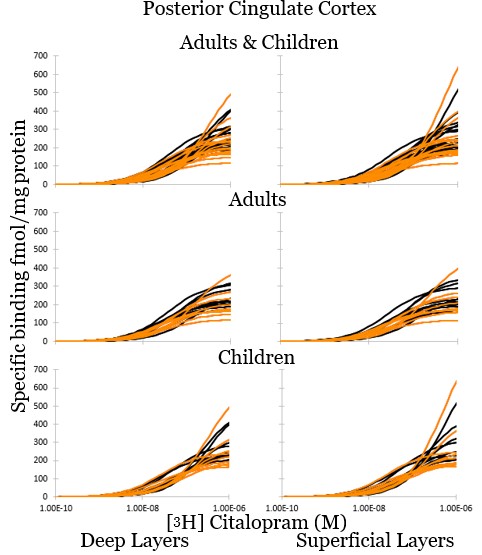

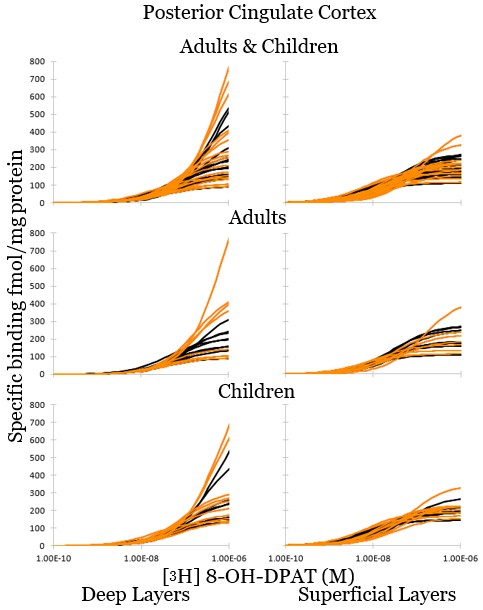
Figure S1. Individual saturation binding curves for all cases in the PCC. Individual curves each include seven concentrations of ligand. Autism (orange) cases had no significantly different binding or affinity for 5-HTT ([3H] citalopram), 5-HT2 ([3H] ketanserin) or 5-HT1A ([3H] 8-OH-DPAT) in adults or children.


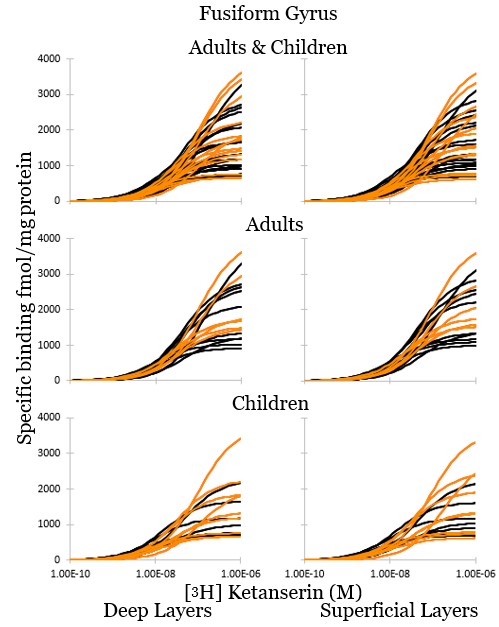


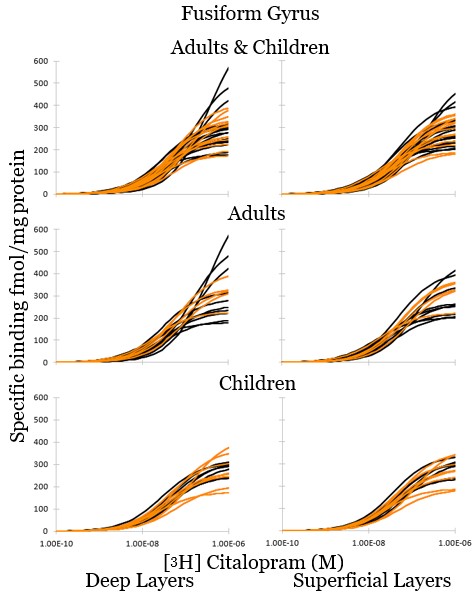

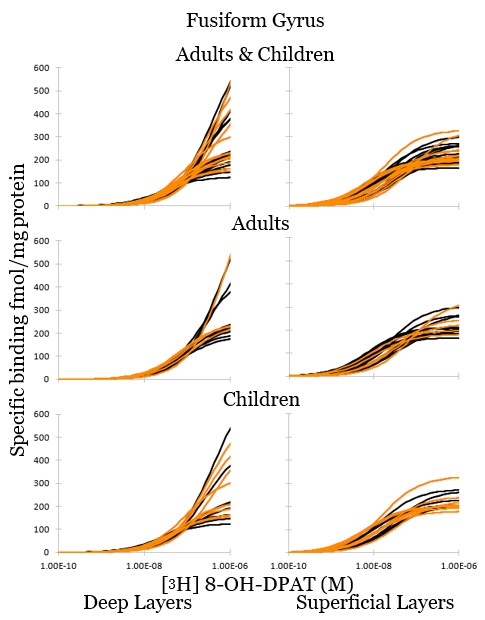
Figure S2. Individual saturation binding curves for all cases in the FG. Individual curves each include seven concentrations of ligand. Autism (orange) cases had no significantly different binding or affinity for

5-HTT ([3H] citalopram), 5-HT2 ([3H]

ketanserin) or 5-HT1A ([3H] 8-OH-DPAT) in adults or children.

Table S1. Bmax and KD for each individual PCC case in both deep and superficial layers. T-tests were performed to compare autism (orange) to controls (black) within groups of adults only, children only and total cases. There were no significantly different group means.

|  | **Posterior Cingulate Cortex** | | | | | |
| --- | --- | --- | --- | --- | --- | --- |
|  | **Deep Layers** | | | **Superficial Layers** | | |
| **CASES** | [3H] Ketanserin | [3H] Citalopram | [3H] 8OH-DPAT | [3H] Ketanserin | [3H] Citalopram | [3H] 8OH-DPAT |
| **Adults**  4103  4104  4267  4268  4271  4275  4345  4599  5813  **Mean**  **±SEM**  **1401**  **1484**  **2825**  **3845**  **4099**  **5574**  **5754**  **5771**  **5864**  **Mean**  **±SEM T-test Children**  4103  4104  4267  4268  4271  4275  4345  4599  5813  **Mean**  **±SEM**  **3871**  **3924**  **4334**  **4721**  **4849**  **5144**  **5302**  **5308**  **5565**  **5841**  **Mean**  **±SEM T-test**  **T-test all cases control vs autism** | Bmax pKD | Bmax pKD | Bmax pKD | Bmax pKD | Bmax pKD | Bmax pKD |
|  | 2884 7.2699  2160 7.5674  936.7 7.901  1843 7.4869  1076 7.7464  886.9 8.043  2329 7.1386  1462 7.6084  1976 7.2103  **1728 7.55**  **229 0.10**  **2017 7.414**  **2764 7.368**  **1832 7.576**  **2351 7.349**  **1537 7.556**  **3033 7.424**  **1164 7.92**  **2350 7.415**  **2968 7.146**  **2224 7.46**  **215 0.07**  **0.1343 0.4873**  3469 6.9727  1332 7.7275  1235 7.87  1779 7.3847  1150 7.7652  1101 7.5941  1688 7.5079  1531 7.5361  2574 7.1956  **1762 7.51**  **261 0.10**  **2076 7.504**  **2626 7.2**  **2057 7.333**  **1636 7.566**  **1108 7.653**  **1810 7.267**  **2327 7.34**  **1241 7.224**  **2196 7.319**  **2875 6.853**  **1995 7.33**  **178 0.07**  **0.4725 0.1422**  **0.1085 0.1127** | 226.4 7.5802  210.1 7.5578339  297.2 7.2858  316.5 7.4493  224 7.4379  337.8 7.1495  193.8 7.5745  182.8 7.392  264.5 6.8807  **250.3 7.37**  **18.7 0.08**  **241.4 7.432**  **182.9 7.401**  **210.7 7.3**  **152.9 7.465**  **173.3 7.62**  **284.4 7.251**  **119.6 7.574**  **422.2 6.79**  **167.1 7.712**  **217.2 7.39**  **30.4 0.09**  **0.3686 0.8270**  293.6 7.3163  259.4 7.0578  213.8 7.2995  531.5 6.4891  251.2 7.0447  504.8 6.6306  260.4 7.1035  231.8 7.5591  351 6.7783  **321.9 7.03**  **39.36 0.12**  **168.8 7.637**  **203.4 7.3**  **653.6 6.486**  **242.4 7.041**  **183.8 7.399**  **244.6 7.583**  **375.1 6.711**  **196.1 7.106**  **282.6 6.919**  **283.4 7.13**  **50.8 0.13**  **0.5573 0.5710**  **0.3467 0.5832** | 142.4 7.1875  162.7 7.3225  268.5 6.9355  169.3 7.1024  91.51 7.4394  256.2 7.0435  377.7 6.6521  202.6 7.3922  216.4 7.1182  **209.7 7.13**  **28.0 0.08**  **108.9 7.347**  **489.8 6.609**  **1394 6.07**  **148.2 7.329**  **105.1 7.497**  **416.6 6.767**  **94.87 7.389**  **480.9 6.74**  **226.5 6.971**  **385 6.97**  **137.4 0.16**  **0.2440 0.3667**  155.7 7.4435  806.8 6.2328  142.8 7.2539  775.3 6.3347  170.1 7.2012  267.8 6.9183  254.3 7.0744  284.4 6.9658  548.7 6.5763  **378.4 6.89**  **87.8 0.14**  **142 7.718**  **184.1 7.189**  **164.7 7.164**  **315.2 7.065**  **222.2 7.244**  **1075 6.233**  **887.7 6.335**  **141.7 7.149**  **291.2 7.089**  **278.2 7.022**  **370.2 7.02**  **104.6 0.14**  **0.9526 0.5117**  **0.3951 0.9124** | 2974 7.2397  2153 7.5381  919 7.9059  1822 7.4943  1014 7.8167  883.5 8.0878  2906 7.1445  1673 7.5331  1868 7.2641  **1801 7.56**  **263 0.11**  **2166 7.344**  **2624 7.404**  **1839 7.603**  **2266 7.381**  **1542 7.545**  **3177 7.333**  **1120 7.916**  **2379 7.402**  **3464 7.045**  **2286 7.44**  **248 0.08**  **0.1987 0.3935**  3037 7.1278  1436 7.6914  893.8 8.1726  2216 7.3161  943.1 7.8887  816.5 7.8468  1981 7.423  1589 7.5033  2545 7.2129  **1717 7.58**  **262 0.12**  **2221 7.469**  **2613 7.22**  **2114 7.322**  **1689 7.539**  **1203 7.596**  **1854 7.236**  **2480 7.326**  **1102 7.273**  **2059 7.363**  **5102 6.503**  **2244 7.28**  **354 0.10**  **0.2490 0.0677**  **0.0804 0.0434** | 233.7 7.5513  205.9 7.590405  336.3 7.1789  297.5 7.5743  222.1 7.4559  360.4 7.1198  196.3 7.5677  174.8 7.4264  260.4 6.8877  **254.2 7.37**  **21.5 0.08**  **251.2 7.399**  **185.6 7.41**  **222.6 7.247**  **164.9 7.402**  **175.4 7.632**  **277.8 7.257**  **117.8 7.598**  **473.9 6.7**  **172.5 7.73**  **226.9 7.38**  **34.8 0.10**  **0.5162 0.9850**  314.3 7.2467  251.6 7.0705  213.3 7.3128  786.3 6.2674  229 7.1178  474 6.667  265.7 7.1127  233.3 7.5693  380.9 6.7371  **349.8 7.01**  **61.4 0.13**  **172.7 7.593**  **227.8 7.194**  **967 6.271**  **258 6.968**  **195.6 7.363**  **252.5 7.558**  **455.1 6.592**  **191 7.144**  **192.3 7.267**  **323.6 7.11**  **85.3 0.15**  **0.8062 0.6362**  **0.6426 0.7046** | 188.9 7.4987  189.1 7.6838  273.6 7.5262  162.6 7.8444  109.7 8.0893  278.1 7.5058  246.1 7.4791  253.8 7.6341  178.3 7.8677  **208.9 7.68**  **19.0 0.07**  **135.7 8.14**  **225.3 7.454**  **433.1 6.857**  **167.1 7.967**  **115.9 8.257**  **188.5 7.509**  **116.9 8.064**  **246.1 7.535**  **164.5 7.787**  **199.2 7.73**  **32.8 0.15**  **0.8028 0.7660**  204 7.8864  274.6 7.308  150 8.0053  185.8 8.0121  142.3 8.2674  218.6 7.5732  187.4 7.6874  195.5 7.7238  227.3 7.699  **198.4 7.80**  **13.4 0.09**  **188.1 8.163**  **168.5 7.924**  **234.4 7.323**  **224.1 7.528**  **167.1 8.224**  **227.4 7.766**  **347.3 7.19**  **157.4 7.503**  **205.3 7.753**  **227.1 7.654**  **214.7 7.70**  **17.3 0.11**  **0.4665 0.5254**  **0.8605 0.8321** |

Table S2. Bmax and KD for each individual FG case in both deep and superficial layers. T-tests were performed to compare autism (orange) to controls (black) within groups of adults

only, chidren only and total cases. There were no significantly different group means.

|  | **Fusiform Gyrus** | | | | | |
| --- | --- | --- | --- | --- | --- | --- |
|  | **Deep Layers** | | | **Superficial Layers** | | |
| **CASES** | [3 H] Ketanserin | [3 H] Citalopram | [3 H] 8OH-DPAT | [3 H] Ketanserin | [3 H] Citalopram | [3 H] 8OH-DPAT |
| **Adults**  602  1026  1365  4345  4599  4605  4916  5321  5813  5873  6004  **Mean**  **±SEM**  **5027**  **5574**  **5771**  **5864**  **6337**  **Mean**  **±SEM T-test Children**  1714  4670  5170  5242  5334  5387  5408  **Mean**  **±SEM**  **2004**  **3924**  **4334**  **4899**  **5144**  **5302**  **5308**  **5565**  **5841**  **Mean**  **±SEM T-test**  **T-test all cases control vs autism** | Bmax pKD | Bmax pKD | Bmax pKD | Bmax pKD | Bmax pKD | Bmax pKD |
|  | 1188 7.91  2132 7.54  2782 7.26  1236 7.35  919.7 7.88  2812 7.38  1033 7.96  2620 7.37  3787 6.80  1361 7.56  1715 7.59  **1962 7.51**  **281 0.10**  **3949 7.01**  **1514 7.63**  **1472 7.46**  **1802 7.32**  **3228 7.00**  **2393 7.28**  **504 0.13**  **0.4814 0.2176**  772.9 7.94  999.2 7.62  723.9 8.26  1671 7.82  2267 7.34  701.9 8.18  1188 8.01  **1189 7.88**  **221 0.12**  **1991 6.98**  **2269 7.48**  **661.1 8.11**  **1871 7.59**  **678.4 7.87**  **3747 7.01**  **762.1 8.07**  **1358 7.46**  **1204 7.62**  **1616 7.58**  **332 0.14**  **0.3040 0.1264**  **0.5193 0.1877** | 229.7 7.47  544.0 6.85  260.5 7.31  769.4 6.44  287.3 7.43  239.9 7.57  322.5 7.51  179.9 7.85  489.1 6.79  194.0 7.46  **351.6 7.27**  **60.1 0.14**  **340.9 7.37**  **411.8 7.19**  **336.1 7.06**  **338.6 7.21**  **227.9 7.70**  **331.1 7.31** | 205.2 7.08  191.2 7.07  266.2 6.91  822.0 6.23  238.0 7.06  570.8 6.41  261.5 6.80  239.8 7.06  457.7 6.67  230.5 6.95  222.7 6.92  **336.9 6.83**  **60.1 0.09**  **986.5 6.07**  **246.2 7.14**  **254.0 7.02**  **245.8 7.11**  **243.0 6.92**  **395.1 6.85** | 1177 7.93  2271 7.51  2549 7.31  1384 7.27  1004 7.79  2933 7.36  1094 7.93  2644 7.41  3501 6.88  1364 7.59  1774 7.57  **1972 7.50**  **256 0.09**  **3907 7.04**  **1538 7.66**  **1631 7.41**  **2171 7.18**  **2862 7.10**  **2422 7.28**  **440 0.11**  **0.4071 0.1764**  923.7 7.70  1060 7.58  741.7 8.22  1625 7.86  2229 7.34  694.6 8.19  1181 8.04  **1208 7.85**  **207 0.12**  **2867 6.74**  **2461 7.41**  **739.5 7.95**  **1945 7.58**  **624.8 7.98**  **3596 7.06**  **791.4 8.05**  **1376 7.46**  **1337 7.50**  **1749 7.53**  **348 0.14**  **0.2053 0.1259**  **0.3631 0.1368** | 222.7 7.55  356.2 7.19  269.1 7.30  545.2 6.70  270.8 7.51  259.4 7.54  412.2 7.31  203.7 7.83  475.8 6.83  215.0 7.37  **323.0 7.31**  **37.6 0.11**  **380.6 7.25**  **375.6 7.24**  **348.5 7.06**  **345.2 7.22**  **224.8 7.73**  **334.9 7.30**  **28.4 0.11**  **0.8043 0.9380**  315.5 7.33  247.4 7.39  309.7 7.36  341.6 7.44  312.3 7.11  329.3 7.16  238.8 7.44  **299.2 7.32**  **15.1 0.05**  **247.6 7.44**  **190.7 7.61**  **282.4 7.38**  **189.2 7.30**  **358.4 7.26**  **310.5 7.42**  **280.5 7.30**  **380.6 6.94**  **280 7.33**  **24.8 0.07**  **0.5210 0.8967**  **0.6893 0.9766** | 269.2 7.36  200.3 7.52  268.9 7.62  188.2 8.04  167.5 8.12  206.0 7.67  214.0 8.12  305.3 7.62  209.4 8.09  190.2 7.69  182.6 7.93  **218.3 7.80**  **13.1 0.08**  **328.9 7.12**  **248.9 7.54**  **198.6 8.08**  **182.3 7.96**  **225.8 7.55**  **236.9 7.65**  **25.7 0.17**  **0.5424 0.3863**  208.7 7.97  207.1 7.58  275.5 7.82  269.0 7.51  199.3 7.68  229.9 7.76  205.4 8.00  **227.8 7.76**  **12.0 0.07**  **195.8 7.89**  **179.9 7.95**  **242.8 7.69**  **223.3 7.46**  **330.6 7.79**  **202.5 8.19**  **209.6 7.81**  **206.9 7.84**  **201.7 8.12**  **221.5 7.86**  **14.8 0.07**  **0.7432 0.3457**  **0.7555 0.9794** |
|  |  | **29.4 0.11 147.9 0.20**  **0.7638 0.8614 0.7292 0.9248**  304.6 7.35 153.8 7.34  252.7 7.35 211.8 6.98  325.6 7.34 126.6 7.60  308.0 7.55 744.0 6.41  295.3 7.17 243.4 6.92  316.8 7.19 474.7 6.58  246.3 7.44 168.2 7.41  **292.8 7.34 303.2 7.03**  **11.7 0.05 85.5 0.17**  **254.9 7.41 224.1 7.11**  **169.4 7.31** | |  |  |  |
|  |  | **175.9 7.62**  **264.3 7.46**  **204.7 7.22**  **367.7 7.23**  **316.2 7.42**  **271.6 7.28**  **423.7 6.89**  **284.9 7.32**  **28.9 0.08**  **0.8063 0.7938**  **0.5565 0.9003** | **199.0 7.24**  **477.9 6.45**  **327.5 7.01**  **528.9 6.56**  **230.7 6.99**  **158.7 7.39**  **607.7 6.52**  **324.9 6.95**  **56.7 0.12**  **0.8368 0.6967**  **0.7398 0.9641** |  |  |  |


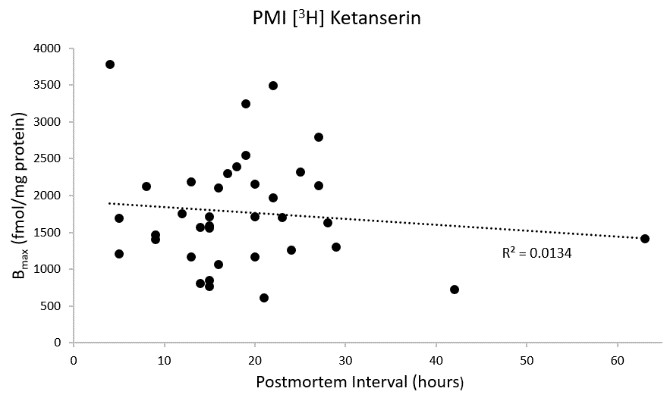


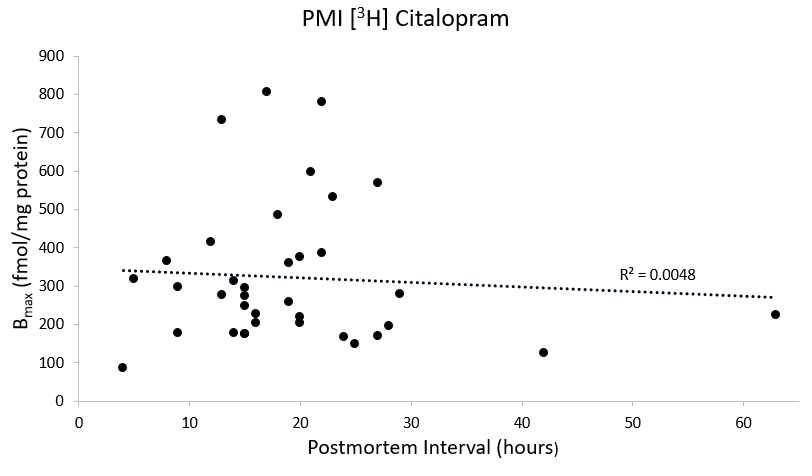
Figure S3. Postmortem interval (PMI) vs receptor binding. There was no significant correlation of PMI with serotonin receptor binding in the anterior cingulate cortex. Therefore, significant changes in receptor number in autism compared to controls is not explained by increased PMI.

References

Buckner R. L., Andrews-Hanna J. R. and Schacter D. L. (2008) The

Brain’s Default Network: anatomy function, and relevance to

disease. *Ann. N. Y. Acad. Sci.* **1124**, 1–38.

Greicius M. D., Krasnow B., Reiss A. L. and Vinod Menon (2003)

Functional connectivity in the resting brain: a network analysis of

the default mode hypothesis*. Proc. Natl. Acad. Sci. USA* **100**, 253–

258.

Kanwisher N., McDermott J. and Chun M. M. (1997) The fusiform face

area: a module in human extrastriate cortex specialized for face

perception. *J. Neurosci*. **17**, 4302–4311.

Kennedy D. P. and Courchesne E. (2008) The intrinsic functional

organization of the brain is altered in autism. *NeuroImage* **39**,

1877–1885.

Oblak A. L., Gibbs T. T. and Blatt G. J. (2010) Decreased GABAB

receptors in the cingulate cortex and fusiform gyrus in Autism. J.

*Neurochem*. **114**, 1414–1423.

Oblak A., Gibbs T. T. and Blatt G. J. (2011a) Reduced GABAA receptors

and benzodiazepine binding sites in the posterior cingulate cortex and

fusiform gyrus in autism. *Brain Res.* **1380**, 218–228.

Oblak A. L., Rosene D. L., Kemper T. L., Bauman M. L. and Blatt G. J.

(2011b) Altered posterior cingulate cortical cytoarchitecture, but

normal density of neurons and interneurons in the posterior

cingulate cortex and fusiform gyrus in autism*. Autism Res*. **4**, 200–

211.

Oblak A., Gibbs T. T. and Blatt G. J. (2013) Reduced serotonin receptor

subtypes in a limbic and a neocortical region in autism. *Autism Res.*

**6**, 571–583.

Pierce K. and Redclay E. (2008) Fusiform function in children with an

ASD is a matter of “who”. *Biol. Psychiatry* **64**, 552–560.

Pierce K., M€uller R. A., Ambrose J., Allen G. and Courchesne E. (2001)

Face processing occurs outside the fusiform ‘face area’ in autism:

evidence from functional MRI*. Brain* **124**, 2059–2073.

Schultz R. T., Grelotti D. J., Klin A., Kleinman J., Van der Gaag C.,

Marois R. and Skudlarski P. (2003) The role of the fusiform face

area in social cognition: implications for the pathobiology of

autism. Philos. *Trans. R Soc. Lond. B Biol. Sci*. **358**, 415–427.

Van Kooten I. A. J., Palmen S. J. M. C., von Cappeln P., Steinbusch H.

W. M., Korr H., Heinsen H., Hof P. R., van Engeland H. and

Schmitz C. (2008) Neurons in the fusiform gyrus are fewer and

smaller in autism. *Brain* **131**, 987–999.

Weng S. J., Wiggins J. L., Peltier S. J., Carrasco M., Risi S., Lord C. and

Monk C. S. (2010a) Alterations of resting state functional

connectivity in the default network in adolescents with autism

spectrum disorders. *Brain Res.* **1313**, 202–214.

Weng S. J., Carrasco M., Swartz J. R., Wiggins J. L., Kurapati N.,

Liberzon I., Risi S., Lord C. and Monk C. S. (2010b) Neural

activation to emotional faces in adolescents with autism spectrum

disorders*. J. Child Psychol. Psychiatry* **52**, 296–305.
